# The contribution of mechanical ventilation to critical illness-associated diaphragm dysfunction: longitudinal trajectories alone do not establish causal primacy

**DOI:** 10.1186/s13054-026-06308-y

**Published:** 2026-09-05

**Authors:** Xibin Feng, Miao Yang, Wei Xu

**Affiliations:** Pediatric Intensive Care Unit, National Children’s Medical Center, Shengjing Hospital of China Medical University, Shenyang, Liaoning China

## Main text

To the Editor,

Rosà et al. provide a timely critique of treating mechanical ventilation as the main driver of diaphragm weakness in ventilated patients [1]. The review convincingly shows that ventilator-induced diaphragm dysfunction is too narrow as an umbrella term and that critical illness can contribute substantially to early diaphragm weakness. Our concern is with the stronger causal inference. The review acknowledges that the clinical contribution of ventilation is difficult to isolate and that no study in ventilated patients has successfully done so, yet later describes critical illness as the “primary driver” and concludes that diaphragm dysfunction is “primarily a manifestation of critical illness rather than a direct consequence of mechanical ventilation” [1]. The evidence presented supports an important and sometimes early contribution of critical illness, but it does not establish that this contribution generally exceeds that of ventilatory management. This distinction is clinically relevant because the proposed etiological hierarchy contributes to the review’s discussion of whether diaphragm-protective targets should be deprioritised when they conflict with other patient-centred goals. Observed diaphragm trajectories under mechanical ventilation are net outcomes of simultaneous disease, recovery, loading, unloading, and treatment processes; they are not estimates of the effect of one ventilatory strategy compared with another.

Early weakness does not quantify the effect of subsequent ventilation. Demoule et al. found low stimulated pressure in 64% of patients studied within 24 h after intubation, with sepsis and illness severity associated with lower strength [2]. This establishes that critical illness can contribute before prolonged ventilatory exposure. However, the day-1 measurement was neither a pre-ventilation baseline nor an estimate of what would have occurred under an alternative subsequent strategy; all participants had already been selected for and exposed to invasive ventilation. Conversely, the time-related loss of twitch pressure and structural injury described during controlled ventilation [3], and bidirectional thickness changes associated with low or excessive effort [4], cannot isolate ventilation from evolving disease. The same interpretive limitation therefore applies in both directions.

Recovery during ventilation is likewise not evidence that ventilatory management made no contribution. In Lecronier et al., stimulated pressure increased by 19% in septic patients but decreased by 7% in non-septic patients over several days of ventilation [5]. In the ultrasound subgroup, end-expiratory thickness decreased in both groups. These discordant trajectories are compatible with concurrent mechanisms, for example, recovery of reversible contractile depression while structural remodelling continues, but they do not identify their relative contributions. Most importantly, improvement under exposure is a net change, not a treatment effect: without comparison with a feasible alternative ventilatory strategy, the direction and magnitude of the effect of the observed strategy cannot be determined.

For causal analyses, ventilatory management is better conceptualised as a time-varying strategy rather than a binary exposure. Mode and level of assistance, positive end-expiratory pressure, patient effort, synchrony, and duration of exposure change over time in relation to disease severity, sedation, neuromuscular blockade, and evolving diaphragm and lung physiology. Indeed, the review itself juxtaposes two sepsis models in which controlled ventilation had opposite effects: attenuation of early sarcolemmal injury in a short endotoxaemia protocol [6] and potentiation of diaphragm dysfunction in a longer protocol [7]. These experiments do not establish the corresponding human effects; they support context-dependent effects of unloading rather than a general etiological hierarchy.

The muscle-hibernation findings also support caution. Van den Berg et al. found myosin trapped in an energy-sparing super-relaxed state in diaphragm fibres from ventilated critically ill patients; ancillary quadriceps and ventilated-rat experiments suggested that the abnormality was preferentially observed in the diaphragm rather than being a manifestation of generalised critical illness [8]. This diaphragm-predominant signal does not identify mechanical ventilation as the cause and does not resolve the relative contributions of critical illness and ventilatory management. It therefore supports causal uncertainty rather than either etiological attribution and suggests a candidate diaphragm-specific mechanism whose interpretation may depend on timing, inflammatory state, loading history, and other co-exposures.

Future studies should first specify the causal contrast. A clinically useful question would be: among patients at a defined disease phase, what is the effect of a specified ventilatory strategy, compared with a feasible alternative, over a defined time window, on stimulated pressure, diaphragm structure, and patient-centred outcomes? Randomised strategy trials are preferable when feasible. When causal effects are estimated from observational data, target-trial principles [9] and methods addressing time-varying confounding affected by prior ventilatory management [10] may help align the analysis with a clinically meaningful strategy contrast. Serial assessment remains essential because strength, thickness, effort, and molecular state can move in different directions.

We therefore support critical illness-associated diaphragm dysfunction as umbrella terminology while retaining uncertainty about the relative causal contributions of critical illness and ventilatory management. Early dysfunction and recovery during ventilation establish timing and heterogeneity, not a general etiological hierarchy. The more actionable question is when and in whom ventilatory unloading is protective, and when disuse or overload becomes harmful; longitudinal trajectories alone do not establish causal primacy.

## Data Availability

No datasets were generated or analysed during the current study.
